# Investigating linkage rates among probabilistically linked birth and hospitalization records

**DOI:** 10.1186/1471-2288-12-149

**Published:** 2012-09-25

**Authors:** Jason P Bentley, Jane B Ford, Lee K Taylor, Katie A Irvine, Christine L Roberts

**Affiliations:** 1 Clinical and Population Perinatal Health Research, Kolling Institute of Medical Research, Sydney Medical School, University of Sydney at Royal North Shore Hospital, Building 52, St Leonards, NSW, 2006, Australia; 2 Centre for Epidemiology and Evidence, NSW Ministry of Health, Locked Bag 961, North Sydney, NSW, 2059, Australia; 3 Centre for Health Record Linkage, Cancer Institute, Australian Technology Park, 8 Central Avenue, Eveleigh, NSW, 2015, Australia

**Keywords:** Probabilistic record linkage, Pregnancy, Administrative health data, International classification of diseases

## Abstract

**Background:**

With the increasing use of probabilistically linked administrative data in health research, it is important to understand whether systematic differences occur between the populations with linked and unlinked records. While probabilistic linkage involves combining records for individuals, population perinatal health research requires a combination of information from both the mother and her infant(s). The aims of this study were to (i) describe probabilistic linkage for perinatal records in New South Wales (NSW) Australia, (ii) determine linkage proportions for these perinatal records, and (iii) assess records with linked mother and infant hospital-birth record, and unlinked records for systematic differences.

**Methods:**

This is a population-based study of probabilistically linked statutory birth and hospital records from New South Wales, Australia, 2001-2008. Linkage groups were created where the birth record had complete linkage with hospital admission records for both the mother and infant(s), partial linkage (the mother only or the infant(s) only) or neither. Unlinked hospital records for mothers and infants were also examined. Rates of linkage as a percentage of birth records and descriptive statistics for maternal and infant characteristics by linkage groups were determined.

**Results:**

Complete linkage (mother hospital record – birth record – infant hospital record) was available for 95.9% of birth records, partial linkage for 3.6%, and 0.5% with no linked hospital records (unlinked). Among live born singletons (complete linkage = 96.5%) the mothers without linked infant records (1.6%) had slightly higher proportions of young, non-Australian born, socially disadvantaged women with adverse pregnancy outcomes. The unlinked birth records (0.4%) had slightly higher proportions of nulliparous, older, Australian born women giving birth in private hospitals by caesarean section. Stillbirths had the highest rate of unlinked records (3-4%).

**Conclusions:**

This study shows that probabilistic linkage of perinatal records can achieve high, representative levels of complete linkage. Records for mother’s that did not link to infant records and unlinked records had slightly different characteristics to fully linked records. However, these groups were small and unlikely to bias results and conclusions in a substantive way. Stillbirths present additional challenges to the linkage process due to lower rates of linkage for lower gestational ages, where most stillbirths occur.

## Background

The ability to conduct linkage of perinatal records, obtained as part of routinely collected administrative health data, has increased the scope for population based studies of mother and infant health [1]. When a unique identifier is available, deterministic linkage is used to identify records for the same person [2,3], however, when no unique identifiers are available, increasingly large databases are being linked using probabilistic-based linkage methods. While probabilistic linkage usually involves combining records for individuals, perinatal research typically requires a combination of information from both the mother and her infant(s).

Advantages of linkage of administrative health records include; describing the total disease burden in a population, assessment of risk factors [4] and investigating rare outcomes [5], which are all relevant to addressing key issues in health and health policy [6,7]. Other advantages include; improved coverage, ascertainment [8], completeness and validity [4], and large samples with standardized reporting to produce generalisable results [9]. Longitudinal record linkage allows the study of recurrence risk [10-12], mortality, major morbidities [13] and co-morbidities and impacts on childhood development [14]. Probabilistic linkage of administrative health records is undertaken routinely in Scotland [15], Wales [16,17], Canada [18-20], the United States [21], and Australia [22,23].

Mismatches are possible with probabilistic linkage. Two different individuals could be linked resulting in incorrectly reported outcomes or risk factors (false positive links), or two records from the same individual may not be linked (false negative links), resulting in missing information. The success of linkage, often described in terms of minimizing mismatches, can depend upon a number of factors, including the quality of the information used in the linkage process and how uniquely identifying reported information is. Recent studies have shown that, unlike deterministic methods, the flexibility of probabilistic record linkage allows for minimization of mismatches under variations in data quality [24]. With the potential for mismatches it is important to consider the possibility of systematic biases that may arise between linked and unlinked populations of records. Researchers are becoming increasingly aware of the potential bias created by excluding unlinked records, and more recently this has prompted a publication of guidelines for reporting studies using linked data [25].

The aims of this study were to (i) describe probabilistic linkage for perinatal records in New South Wales (NSW) Australia, (ii) determine linkage proportions for these perinatal records, and (iii) assess records with complete linkage of mother and infant hospital-birth record and unlinked records for systematic differences.

## Methods

### Data sources

This study used linked records of the NSW Perinatal Data Collection (PDC), and the NSW Admitted Patient Data Collection (APDC). The PDC (referred to as ‘birth records’) is a population-based statutory surveillance system that includes all live births and stillbirths of at least 20 weeks gestation or if gestational age is not known of at least 400 grams birth weight, and includes information on maternal characteristics, pregnancy, labor and delivery factors and infant outcomes. ‘Hospital records’ (for mothers and infants) that relate to the birth (birth admission records) were obtained from the APDC, which includes demographic and hospitalization related data for every inpatient admitted to any public or private hospital in NSW. Diagnoses and procedures for each hospital admission are coded according to the 10^th^ revision of the International Classification of Disease, Australian Modification (ICD10-AM) and the Australian Classification of Health Interventions (ACHI).

### Study population

The study population included all mothers who gave birth, and their infants, in NSW, Australia, from 1 January 2001-31 December 2008. NSW is the largest state in Australia with around 7,287,600 million people representing 32% of the Australian population [26]. Homebirths (0.2%) as identified in the birth records were excluded as these would not have a linked hospital birth admission.

### Probabilistic record linkage

Birth, and maternal and infant hospital records for 2001 to 2008 were probabilistically linked [27] by the Centre for Health Record Linkage (CHeReL) [23] using a best practice approach in privacy preserving record linkage [28] and the open source probabilistic record linkage software Choice Maker [29]. Best practice involves ensuring separation of personal identifiers and health information. The CHeReL receives personal identifiers only (i.e. no health information) from the data custodians to generate a linkage key, and a linkage key is returned to the data custodians. Finally, researchers receive only health information and a linkage key from the data custodians.

The link between the mother and infant is provided by the common birth record. Probabilistic linkage is used to link records for the same individual, and in this context the outline that follows is in reference to linking the mothers’ birth and hospital records, and the infants’ birth and hospital records.

The CHeReL used a variety of fields that are common to both datasets for matching records in the linkage process. These include first name, last name, address, sex, date of birth, and country of birth. Additional information used, where available, includes hospital code and medical record number (MRN), admission date, discharge date, hospital discharged from, hospital discharged to, alias names, plurality and birth order for multiple pregnancies (twins, triplets and higher order multiple pregnancies).

**Figure 1:**
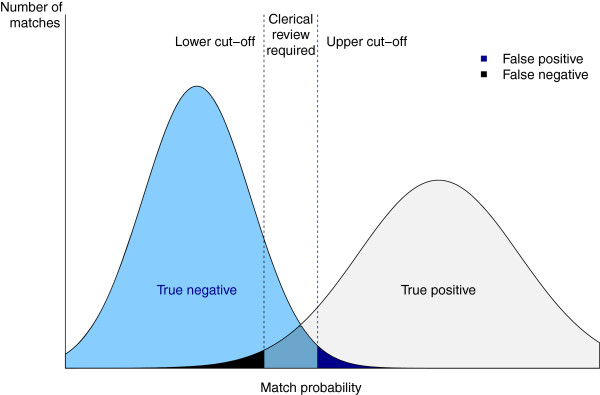
**Use of thresholds to minimise false positive and negative matched records**. In this theoretical example the two vertical lines show the lower and upper cut-offs (thresholds) that are used in probabilistic record linkage to accept or reject matches. The amount of false positive and negative matches can be managed by moving the thresholds. If false matches are unacceptably high the upper cut-off can be moved to the right, creating more clerical reviews and minimising false decisions from automatically accepting records as a match. A similar process can be used to minimise false negatives.

Standardization and parsing techniques are used to allow a comparison of common fields and to facilitate matching. As a first stage, blocking is used to quickly search the target database for records that are possible matches. ‘Blocking’ is an automated algorithm designed to find as many as possible records that potentially match each other without exceeding a given and manageable block size. This increases the efficiency of a second stage of more detailed matching by reducing the number of pairs that are compared in the more accurate second stage matching. Records within the same block are scored during the second stage of matching. ‘Scoring’ generates the probability that two records match based on a series of weighted ‘clues’. Clues (known as ‘features’ in Artificial Intelligence literature) are attributes of records that are suggestive of match or non-match decisions. Examples of clues are that the date of birth does not match, or there is a match on the phonetic code for the first name. Phonetic code is generated from coding schemes such as Soundex and the New York State Identification and Intelligence System (NYSIIS). This reduces the effect of minor typographical errors or spelling variations by assigning the same codes to words or syllables with similar pronunciation i.e. Robert and Rupert. The weight for each clue has been derived using previously matched data and a machine learning process called Maximum Entropy Modeling. During the scoring process these weights are combined using a formula based on maximum entropy theory to create a probability between 0 and 1 that two records match. Upper and lower probability cut-offs (thresholds) determine whether records are classified as matches, non-matches, or possible matches requiring clerical review (Figure 1). The CHeReL initially uses upper and lower probability cut-offs of 0.75 and 0.25 and adjusts these manually for each individual linkage to minimize false and missed links. Groups of records with indeterminate probabilities are reviewed manually to determine whether they should be classified as a match or not.

The CHeReL undertakes quality assurance for any data linkage and assesses the linkage quality by manually reviewing personal identifiers for a sample of the records obtained for linkage. For this project, the CHeReL reported the linkage quality as < 1/1,000 missed links and < 2/1,000 false positive links.

### Linkage groups

For this study we defined six different groups of records based on the linkage configuration. The ‘linked mothers and infants’ group includes birth records with a linked hospital admission for both the mother and the infant(s), representing the ‘complete’ set of perinatal records. The ‘mothers only’ group includes birth records with a linked hospital birth admission record for the mother but without one for the infant, while the ‘infants only’ group includes birth records with a linked hospital birth admission record for the infant but without one for the mother. These two groups represent the ‘partial linkage’ groups. Finally, there are three different groups of unlinked records. The first is ‘unlinked birth records’ which includes birth records without a linked birth admission record for either the mother or the infant. The second is the ‘unlinked maternal hospital records’ which includes hospital birth admission records identified for a pregnancy that did not link to the birth records. The third is the ‘unlinked infant hospital records’ which includes hospital birth admission records identified for infants that did not link to the birth record.

### Stillbirths and plurality

Stillbirths are reported on the mother’s hospital birth admission record and do not usually generate an infant hospital admission record for the infant. Therefore most will not have complete linked mother and infants records. Further, there may be misclassification of stillbirths and miscarriages and it has been indicated previously that linkage for stillbirths is problematic [30].

Linking is conducted separately for singleton and multiple pregnancies as multiple pregnancies generate infant records with identical information such as mothers name, date of birth, hospital of birth and even sex, so extra care is required [31,32].

### Identification of hospital birth admission records

ICD10-AM [33] diagnosis and ACHI procedure codes, and administrative information, were used to identify hospital birth admission records for mothers and infants independently of the birth record.

Infant birth admissions were initially selected where records indicated an age of 0-1 days and either a live birth (ICD10-AM = Z38), born in hospital, or a birth weight and an ICD10-AM code for a condition of the perinatal period. For those records that linked to the birth record, we required the admission date to be within ±1 day of the date of birth and the hospital of birth reported on the hospital record to match that reported on the birth record (Table 1).

**Table 1:** Variables used to identify birth admission hospital record for infants

**Variable**	**Value**	**Description**
ICD10-AM	Z38	Liveborn infants according to place of birth
ICD10-AM	P-codes	Conditions originating in the perinatal period
Calculated age	[0, 0.0028]	An age of 0 or 1 days old
Birth weight	Non-missing	A birth weight is recorded in the hospital record
Source of referral	Born in hospital	The hospital record is for birth in hospital
Admission order	1	The hospital record is the first for an infant

ICD10-AM: 10^th^ revision of the International Classification of Disease, Australian Modification.

Maternal hospital records for the birth admission were initially selected where there were any ICD10-AM diagnosis or procedure codes reported for delivery. We also required the same hospital of birth to be reported by the hospital and birth record, and the date of birth to have occurred during the period between the admission and separation dates for the selected birth admission record (Table 2).

**Table 2:** Variables used to identify delivery admission hospital records for mothers

**Description**	**ICD10-AM/ACHI codes**
Delivery	O80-O84
Outcome of delivery	Z37
Preterm delivery	O60.1-O60.3
Delivery procedures	90467-90470, 16520
Postpartum sutures	16571, 16573, 90479-90481, 90485
Other procedures associated with delivery	90472-90477
Analgesia and anaesthesia during labour and delivery procedure	92506,92507
Induction and augmentation of labour	90465,90466

ICD10-AM diagnosis codes from the International Classification of Disease Version 10, Australian Modification.

ACHI procedure codes from the Australian Classification of Health Interventions.

### Variables

Maternal variables compared between linkage groups were gestation that antenatal care commenced, marital status, country of birth (Australia/other), birth in a private hospital, delivery by caesarean section, diabetes, hypertension, induction of labor, maternal age, parity (number of previous births), smoking during pregnancy, placenta praevia, placental abruption, duration of pregnancy less than 26 weeks gestation and socio-economic status (Australian Bureau of Statistics Socio-Economic Index For Areas – Index of Relative Socio-economic Disadvantage) [34]. Infant variables compared across linkage groups were admission to a special care nursery (SCN) or neonatal intensive care unit (NICU), Apgar score at one minute less than 4, sex, birth weight, death in hospital, and gestational age. All variables, except for marital status, placental abruption and placenta praevia were available from the birth record, and where possible obtained from the hospital birth admission records using diagnosis and procedure codes (Table 3).

**Table 3:** Identification of variables for unlinked hospital records

**Group**	**Description**	**ICD10-AM/ACHI codes**
Infants	Preterm birth	P07.2, P07.3
	Apgar1 < 4	P20.1
Mothers	Diabetes	O24, E10, E11, E13, E14
	Hypertension	O10, O11, O13-O16
	Induction	90465
	Caesarean section	O82, Procedures: 16520
	Placenta praevia	O44.1
	Placental abruption	O45
	Duration of pregnancy < 25 weeks	O90.1, O90.2, O90.3
Singleton		Z37.0-Z37.1, Z38.0-Z38.2, O80-O83
Multiple births		Z37.2-Z37.7, Z38.3-Z38.8, O84
Stillbirth		Z37.1, Z37.3, Z37.4, Z37.6, Z37.7

ICD10-AM diagnosis codes from the International Classification of Disease Version 10, Australian Modification.

ACHI procedure codes from the Australian Classification of Health Interventions.

### Analysis

Reported for all births are (i) rates of linkage for the birth-hospital record linkage groups by plurality and live born/stillborn as a percentage of all birth records and (ii) rates of identification for deliveries and births as ascertained from the hospital birth admissions as a percentage of the number of deliveries/births reported in the birth records. Note that delivery is used to refer to a mother giving birth, and birth to refer to a baby being born. Thereafter, we limited the analysis to live born singleton deliveries/births. Descriptive statistics of both maternal and infant characteristics by linkage groups were reported using either information from the birth or hospital birth record. For those variables reported on both, information from the birth record was used unless the hospital birth admission record was indicated as being more reliable according to validation studies of birth and hospital data [35-37]. Descriptive analysis was performed in SAS 9.2 [38]. Ethical approval was obtained from the NSW Population and Health Services Research Ethics Committee.

## Results

### Linkage rates for all births

**Figure 2:**
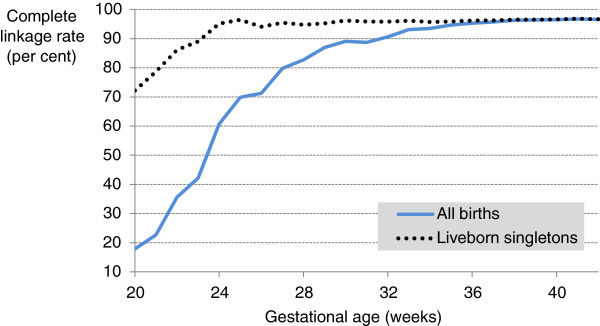
**Linkage rate for complete group by gestational age (weeks).** Complete linkage rate (number of birth records linked to both a mother and infant hospital admission birth record as a percentage of all birth records) by gestational age for all births (blue line) and liveborn singletons (dotted black line).

**Figure 3:**
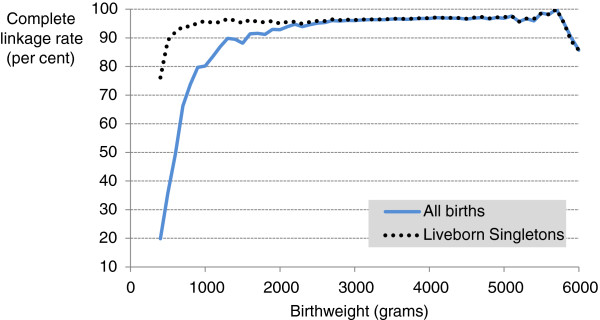
**Linkage rate for complete group by birth weight (grams).** Complete linkage rate (number of birth records linked to both a mother and infant hospital admission birth record as a percentage of all birth records) by birthweight for all births (blue line) and liveborn singletons (dotted black line).

In the period January 2001 to December 2008, there were 706,685 deliveries resulting in 713,522 live births and 4,460 stillbirths recorded in the birth records (PDC). The rate of complete linkage (birth record linked to both mother and infant hospital birth admission records) dropped from around 96% at 37 weeks gestation to <90% at 30 and <70% at 25 (Figure 2). For birth weight, complete linkage was around 95% for weights above 2500 grams, but below this dropped to < 80% by 1000 grams (Figure 3).

Probabilistic linkage resulted in 688,802 birth records with complete linkage to both mother and infant hospital admission birth records (95.9%) (Table 4). Partial linkage was available for a further 3.6% of birth records, including 2.2% with birth record to the mother’s hospital record (’mothers only’) and 1.4% with birth record to the infant’s hospital record (‘infants only’). Less than one per cent (0.5%) of birth records did not link to any hospital record (Table 4).

**Table 4:** Linkage rates for all births, NSW 2001-2008

	**Live birth**		**Stillbirth**		Total^*^
	**Singleton**	**Multiple births**	**Singleton**	**Multiple births**	
	***N*** **= 691 197**	***N*** **= 21 907**	***N*** **= 4 018**	***N*** **= 442**	***N*** **= 717 982**
	***n *(%)**	***n *(%)**	***n *(%)**	***n *(%)**	***n *(%)**
Birth-hospital record linked groups					
Mother and infants	667 315 (96.5)	21 024 (96.0)	60 (1.5)	14 (3.2)	688 802 (95.9)
Mothers only	11 312 (1.6)	502 (2.3)	3 787 (94.3)	414 (93.7)	16 029 (2.2)
Infants only	9 553 (1.4)	314 (1.4)	7 (0.2)	0 (0.0)	9 884 (1.4)
Unlinked groups					
Birth record	3 017 (0.4)	67 (0.3)	164 (4.1)	14 (3.2)	3 267 (0.5)
Infants hospital records	13 469 (-)	620 (-)	- (-)	- (-)	14 504 (-)
Maternal hospital records^†^	8 145 (-)	425 (-)	350 (-)	64 (-)	8 984 (-)

^*^ Total includes those records that could not be classified as either stillbirth/live birth or singleton/multiple births.

^†^ Stillbirth/live birth and plurality could not be identified for 1,505 of the 704,009 deliveries identified in hospital records (0.2%).

From the hospital records, 713,190 infant birth records were identified, almost the same number of live born birth admissions as reported in the birth records (N = 713,522), > 99.9%. From the hospital records, 704,009 delivery records (mothers) were identified, representing 99.6% of those reported in the birth records (N = 706,906).

For the largest group of birth records, live born singletons, 96.5% of records had complete linkage to both a mother and an infant birth admission record compared to 96.0% of live born multiple births. For stillbirths, the largest linkage group was the ‘mothers only’ at around 94% for both singletons and multiple births. Unlinked birth records were more common for stillbirths (3-4%) than live births (0.3-0.4%).

Given the incomplete linkage of stillbirths (recorded as a maternal outcome) and the difficulty of presenting results for multiple births (requiring duplication of maternal information), comparisons of maternal and infant linkage groups are presented for singleton live births. Coding of stillbirth/live birth and plurality could not be identified for 1,505 of the 704,009 deliveries identified in the hospital records (0.2%) and pregnancies with duration <26 weeks were over-represented in this group (3.2%). Similarly, 415 infant birth admissions (<0.1%) could not be classified and preterm birth was over-represented in this group (6.4%).

### Singleton live births

Among singleton live births the rate of complete linkage dropped from around 96% at 25 weeks gestation to only 72% at 20 weeks gestation (Figure 2). For birth weight, complete linkage was around 96% for weights above 1000 grams, but below this dropped to around 80% by 400 grams (Figure 3).

Maternal characteristics differed across the groups of linked and unlinked records (Table 5). The two groups that appeared most different were the unlinked birth records and the mothers only group. The unlinked birth records had higher proportions of nulliparous, Australian-born women, aged 35 and over, births in private hospitals, by caesarean section and the lowest levels of social disadvantage (quintile 1). Missing health information was more common in the unlinked groups.

**Table 5:** Maternal demographic and birth-related characteristics by linkage group for liveborn singleton pregnancies, NSW 2001-2008

**Variable**	**Birth-hospital record linked groups**			**Unlinked groups**	
	**Mothers and infants**	**Mothers only**	**Infants only**	**Birth records**	**Hospital records**
	***N*** **= 667315**	***N =*** **11312**	***N*** **= 9553**	***N =*** **3017**	***N =*** **8145**
	***n *(%)**	***n *(%)**	***n *(%)**	***n *(%)**	***n *(%)**
Maternal age					
< 25	122 417 (18.3)	2 837 (25.1)	1 870 (19.6)	418 (13.9)	1 691 (20.7)
26-34	407 468 (61.1)	6 418 (56.7)	5 779 (60.5)	1 916 (63.5)	4 753 (58.3)
35+	137 304 (20.6)	2 055 (18.2)	1 851 (19.4)	679 (22.5)	1 716 (21.0)
Marital status (Married)	546 152 (81.8)	8 167 (72.2)	- (-)	- (-)	6 343 (77.7)
Parity					
0	277 713 (41.6)	5 077 (44.9)	3 994 (41.8)	1 378 (45.7)	- (-)
1	224 843 (33.7)	3 561 (31.5)	2 879 (30.1)	976 (32.4)	- (-)
2	102 521 (15.4)	1 533 (13.6)	1 410 (14.7)	390 (12.9)	- (-)
3+	61 127 (9.2)	1 123 (9.9)	1 248 (13.1)	255 (8.5)	- (-)
Australian born mother	478 317 (71.7)	7 458 (65.9)	6 847 (71.7)	2 233 (74.0)	5 740 (70.3)
Social disadvantage					
1 (Least)	140 069 (21.0)	2 711 (24.0)	1 835 (19.2)	919 (30.5)	2 044 (25.1)
2-4	367 930 (55.1)	5 596 (49.5)	5 143 (53.8)	1 463 (48.5)	4 084 (50.1)
5 (Greatest)	158 193 (23.7)	2 918 (25.8)	2 483 (26.0)	592 (19.6)	2 030 (24.9)
Smoked during pregnancy	95 866 (14.4)	2 225 (19.7)	1 904 (19.9)	374 (12.4)	- (-)
Antenatal care ≥ 15 weeks	164 940 (24.7)	3 416 (30.2)	2 598 (27.2)	616 (20.4)	- (-)
Diabetes	34 760 (5.2)	531 (4.7)	428 (4.5)	115 (3.8)	385 (4.7)
Hypertension	50 582 (7.6)	760 (6.7)	490 (5.1)	157 (5.2)	551 (6.8)
Placental abruption	2 555 (0.4)	68 (0.6)	- (-)	- (-)	35 (0.4)
Placenta praevia	3 595 (0.5)	56 (0.5)	- (-)	- (-)	127 (1.6)
Induction of labour	166 647 (25.0)	2 633 (23.3)	2 146 (22.5)	739 (24.5)	1 465 (18.0)
Delivery by caesarean	179 528 (26.9)	3 189 (28.2)	2 028 (21.2)	906 (30.0)	2 136 (26.2)
Duration of pregnancy < 26 weeks	1 166 (0.2)	92 (0.8)	21 (0.2)	13 (0.4)	68 (0.8)
Birth in private hospital	168 036 (25.2)	3 486 (30.8)	1 939 (20.3)	1 731 (57.4)	2 047 (25.1)

The ‘mothers only’ group (no associated infant hospital record), had higher levels of social disadvantage (quintile 5), women aged less than 25, non-Australian born mothers, births by unmarried women, smoking during pregnancy, commencement of antenatal care after 14 weeks gestation, caesarean section, placental abruption, and duration of pregnancy less than 26 weeks.

Infant characteristics also varied across linkage groups (Table 6). The ‘mothers only’ group appeared most different with higher proportions of admission to a SCN or NICU, Apgar score at 1 minute less than 4, birth weight less than 1000 grams, birth less than 37 weeks gestation, and infant deaths in hospital.

**Table 6:** Infant demographic and birth-related characteristics by linkage group for liveborn singleton births, NSW 2001-2008

**Variable**	**Birth-hospital record linked groups**			**Unlinked groups**	
	**Mothers and infants**	**Mothers only**	**Infants only**	**Birth records**	**Hospital records**
	***N*** **= 667315**	***N =*** **11312**	***N*** **= 9553**	***N =*** **3017**	***N =*** **13469**
	***n *(%)**	***n *(%)**	***n *(%)**	***n *(%)**	***n *(%)**
Sex					
Male	343 655 (51.5)	5 782 (51.1)	4 851 (50.8)	1 484 (49.2)	6 867 (51.0)
Female	323 261 (48.4)	5 517 (48.8)	4 698 (49.2)	1 496 (49.6)	6 599 (49.0)
Birthweight < 1000 grams	1 948 (0.3)	118 (1.0)	30 (0.3)	16 (0.5)	89 (0.7)
Preterm birth	35 776 (5.4)	806 (7.1)	621 (6.5)	165 (5.5)	745 (5.5)
Agpar1 < 4	12 642 (1.9)	307 (2.7)	198 (2.1)	48 (1.6)	186 (1.4)
Admission to SCN/NICU	100 498 (15.1)	1 908 (16.9)	1 534 (16.1)	440 (14.6)	- (-)
Death in hospital	1 714 (0.3)	125 (1.1)	36 (0.4)	21 (0.7)	81 (0.6)

*SCN*: Special Care Unit.

*NICU*: Neonatal Intensive Care Unit.

## Discussion

To our knowledge, this is the first study that has assessed the linkage of mother and infant birth and hospital records rather than mothers and infants separately. As maternal and pregnancy factors are important predictors of infant outcomes, assessment of the complete linkage is important. In this study the level of complete linkage (95.9%) was high for all births and highest for live singleton births (96.5%). Partially linked mother records (no infant hospital record) had slightly higher rates of adverse events and common risk factors while the partially linked infant records (no mother hospital record) were very similar to those with complete linkage.

This study has shown that stratifying linkage by plurality to overcome the recognized difficulty of linking multiple births [31,32] has generated comparable linkage rates for singleton and multiple live births. Stillbirths represent a very different group in terms of linkage. As infant hospital admission records are not generated, stillbirths should not be present in the complete linkage group. While this explains the majority of stillbirth records being in the ‘mothers only’ group, the proportion of unlinked birth records for stillbirths was also much greater than that for live births (4% vs. 0.4%), reflecting that stillbirths remain a problem for linkage. The lower rate of linkage for stillbirths and the issue of lower rates of complete linkage for live born singletons ≤24 weeks gestation are probably related. Infants born close to the border of viability (misclassification of stillbirths and live births, and births and miscarriages) have been previously identified as a problematic domain for perinatal record linkage [30]. For these reasons, unless infants ≤24 weeks are of particular interest, studies using probabilistically linked records may benefit from restriction to the population of at least 24 weeks gestation. For stillbirth studies, specialist linkages may be needed to improve linkage rates to the levels needed for robust research.

Among singleton live births, the proportions of birth records with partial (1.4-1.6%) or no linkage (0.4%) to hospital records was small. However, there was some evidence of systematic differences for the partially linked records that had no infant hospitalization record (‘mothers only’). This group has slightly higher rates of adverse infant outcomes and associated risk factors, consistent with observations in other studies [10,39-41]. Reduced matching of infant records may be related to the association between missing information, social disadvantage and adverse outcomes, or that severely ill infants with prolonged hospitalization may not necessarily be coded as a birth admission. Restriction to later gestational ages would further reduce the already small size of this group of records. It is important to quantify the number and characteristics of unlinked or partially linked records to assess the potential for bias in estimation of the burden of disease and association between risk factors and outcomes. In our study inclusion of additional records would not change, for example, the estimated preterm birth rate nor is it likely to change risk estimates. However, in other settings with higher proportions of unlinked or partially linked records, exclusion of such records could introduce bias.

Our finding that the unlinked birth records represent a relatively low risk group of mothers and babies is likely to be a local phenomenon. The over-representation of births in private hospitals in the unlinked birth records is likely a result of missing name information. It is at the discretion of private hospitals as to whether name information is collected, and so generally have a large amount of missing name information for both mothers and infants, thus affecting linkage rates for both mothers and infants. Changes to the data provided from private hospitals for linkage could potentially reduce the size of the unlinked birth records.

The results highlight the importance of comparing the characteristics of probabilistic record linkage for perinatal research for mothers and infants, given the potential bias introduced into analysis by incomplete record linkage. It is recommended that for the chosen study population, linked and unlinked records should be requested for analysis and a comparison of linked and unlinked records be undertaken as part of any research using probabilistically linked data. This is of even greater importance when newly-established datasets and linkages are used, which is in contrast to the well-established datasets and linkage protocols used by the CHeReL which generated the linked data for this study. Further, in order to properly discuss the potential impacts, it is necessary for researchers to have a reasonable understanding of how the probabilistic linkage process works and the matching processes involved.

The hospital birth admission records for mothers and infants that did not link to a birth record were small in number and of comparable size to the number of unlinked birth records, and inevitably include some missed links. However, particularly for mothers, there is difficulty in establishing birth admission records as more than one hospitalization may be identified as a birth admission. Although used in the past [42,43], we found that selecting maternal hospital records on a single outcome of delivery code (ICD10: Z37, ICD9: V27) to be inadequate and a much more comprehensive list was required (Table 2). This agrees with a US study that showed that identifying maternal hospital records using outcome of delivery missed complicated pregnancies [44]. Furthermore, due to the nature of ICD coding there was difficulty in classifying the plurality and whether the birth(s) were live born or stillborn. In general a good understanding of coding practices can help to improve identification of these records.

## Conclusions

Probabilistic methods can achieve high, representative levels of complete linkage for mothers and infants. Although some systematic differences occur for the mothers records that do not link to a corresponding infant record, and to a lesser degree for unlinked birth records with respect to private hospitals, these groups are very small and unlikely to bias estimates of effect or conclusions in a substantive way, particularly if the study population is live born singletons.

## Competing interests

The author(s) declare that they have no competing interests.

## Authors’ contributions

CLR and JBF conceived the project and developed the idea in collaboration with LKT and KAI. All authors (CLR, JBF, JPB, LKT, KAI) contributed to study design, LKT, KAI, CLR and JBF, were responsible for data acquisition and JPB conducted the data analysis. JPB, CLR and JBF initially drafted the manuscript and all authors (CLR, JBF, JPB, LKT, KAI) were involved in critical revision of the intellectual content. All authors (CLR, JBF, JPB, LKT, KAI) approved the final manuscript.

## Pre-publication history

The pre-publication history for this paper can be accessed here:

http://www.biomedcentral.com/1471-2288/12/149/prepub

## References

[B1] Donati S Senatore S Ronconi A Maternal mortality in Italy: a record-linkage study BJOG 2011 118 7 872 879 10.1111/j.1471-0528.2011.02916.x 21392245

[B2] Artama M Gissler M Malm H Ritvanen A Nationwide register-based surveillance system on drugs and pregnancy in Finland 1996-2006 Pharmacoepidemiol Drug Saf 2011 20 7 729 738 10.1002/pds.2159 21626607

[B3] Bonamy AK Parikh NI Cnattingius S Ludvigsson JF Ingelsson E Birth characteristics and subsequent risks of maternal cardiovascular disease: effects of gestational age and fetal growth Circulation 2011 124 25 2839 2846 10.1161/CIRCULATIONAHA.111.034884 22124377

[B4] Stanley FJ Croft ML Gibbins J Read AW A population database for maternal and child health research in Western Australia using record linkage Paediatr Perinat Epidemiol 1994 8 4 433 447 10.1111/j.1365-3016.1994.tb00482.x 7870627

[B5] Bright RA Avorn J Everitt DE Medicaid data as a resource for epidemiologic studies: strengths and limitations J Clin Epidemiol 1989 42 10 937 945 10.1016/0895-4356(89)90158-3 2681546

[B6] Roos LL Nicol JP A research registry: uses, development, and accuracy J Clin Epidemiol 1999 52 1 39 47 10.1016/S0895-4356(98)00126-7 9973072

[B7] Schwartz RM Gagnon DE Muri JH Zhao QR Kellogg R Administrative data for quality improvement Pediatrics 1999 103 1 Suppl E 291 301 9917472

[B8] Roberts CL Algert CS Ford JB Methods for dealing with discrepant records in linked population health datasets: a cross-sectional study BMC Health Serv Res 2007 7 12 10.1186/1472-6963-7-12 17261198 PMC1797010

[B9] Ananth CV Getahun D Peltier MR Salihu HM Vintzileos AM Recurrence of spontaneous versus medically indicated preterm birth Am J Obstet Gynecol 2006 195 3 643 650 10.1016/j.ajog.2006.05.022 16949395

[B10] Adams MM Wilson HG Casto DL Berg CJ McDermott JM Gaudino JA McCarthy BJ Constructing reproductive histories by linking vital records Am J Epidemiol 1997 145 4 339 348 10.1093/oxfordjournals.aje.a009111 9054238

[B11] Savitz DA Stein CR Ye F Kellerman L Silverman M The epidemiology of hospitalized postpartum depression in New York State, 1995-2004 Ann Epidemiol 2011 21 6 399 406 10.1016/j.annepidem.2011.03.003 21549277 PMC3090997

[B12] von Katterfeld B Li J McNamara B Langridge AT Obstetric profiles of foreign-born women in Western Australia using data linkage, 1998-2006 Aust N Z J Obstet Gynaecol 2011 51 3 225 232 10.1111/j.1479-828X.2010.01282.x 21631441

[B13] Parrish KM Holt VL Connell FA Williams B LoGerfo JP Variations in the accuracy of obstetric procedures and diagnoses on birth records in Washington State, 1989 Am J Epidemiol 1993 138 2 119 127 8342530 10.1093/oxfordjournals.aje.a116834

[B14] Liang W Chikritzhs T Obstetric conditions and risk of first ever mental health contact during infancy, childhood and adolescence Midwifery 2012 28 4 379 384 21820779 10.1016/j.midw.2011.06.003

[B15] Kendrick S Clarke J The Scottish Record Linkage System Health Bull (Edinb) 1993 51 2 72 79 8514493

[B16] Centre for Health Information, Research and Evaluation http://www.healthinformaticsresearchlabs.swansea.ac.uk/sailproject.html

[B17] Lyons RA Jones KH John G Brooks CJ Verplancke JP Ford DV Brown G Leake K The SAIL databank: linking multiple health and social care datasets BMC Med Inform Decis Mak 2009 9 3 10.1186/1472-6947-9-3 19149883 PMC2648953

[B18] Record linkage at Statistics Canada http://www.statcan.gc.ca/record-enregistrement/index-eng.htm

[B19] Roos NP Black CD Frohlich N Decoster C Cohen MM Tataryn DJ Mustard CA Toll F Carriere KC Burchill CA A population-based health information system Med Care 1995 33 12 Suppl DS13 20 7500666 10.1097/00005650-199512001-00005

[B20] Chamberlayne R Green B Barer ML Hertzman C Lawrence WJ Sheps SB Creating a population-based linked health database: a new resource for health services research Can J Public Health 1998 89 4 270 273 9735524 10.1007/BF03403934 PMC6990342

[B21] Buehler JW Prager K Hogue CJ The role of linked birth and infant death certificates in maternal and child health epidemiology in the United States Am J Prev Med 2000 19 1 Suppl 3 11 10863124 10.1016/s0749-3797(00)00167-7

[B22] Holman CD Bass AJ Rouse IL Hobbs MS Population-based linkage of health records in Western Australia: development of a health services research linked database Aust N Z J Public Health 1999 23 5 453 459 10.1111/j.1467-842X.1999.tb01297.x 10575763

[B23] Centre for Health Record Linkage http://www.cherel.org.au

[B24] Tromp M Ravelli AC Bonsel GJ Hasman A Reitsma JB Results from simulated data sets: probabilistic record linkage outperforms deterministic record linkage J Clin Epidemiol 2011 64 5 565 572 10.1016/j.jclinepi.2010.05.008 20952162

[B25] Bohensky MA Jolley D Sundararajan V Evans S Ibrahim J Brand C Development and validation of reporting guidelines for studies involving data linkage Aust N Z J Public Health 2011 35 5 486 489 10.1111/j.1753-6405.2011.00741.x 21973256

[B26] Australian Bureau of Statistics. Australian Demographic Statistics 2011 Catalogue 3101.0 http://www.abs.gov.au/AUSSTATS/abs@.nsf/Lookup/3101.0Main+Features1Mar%202011?OpenDocument

[B27] Jaro MA Probabilistic linkage of large public health data files Stat Med 1995 14 5–7 491 498 7792443 10.1002/sim.4780140510

[B28] Kelman CW Bass AJ Holman CD Research use of linked health data–a best practice protocol Aust N Z J Public Health 2002 26 3 251 255 10.1111/j.1467-842X.2002.tb00682.x 12141621

[B29] Open Source ChoiceMaker Technology http://oscmt.sourceforge.net

[B30] Ford JB Roberts CL Taylor LK Characteristics of unmatched maternal and baby records in linked birth records and hospital discharge data Paediatr Perinat Epidemiol 2006 20 4 329 337 10.1111/j.1365-3016.2006.00715.x 16879505

[B31] Meray N Reitsma JB Ravelli AC Bonsel GJ Probabilistic record linkage is a valid and transparent tool to combine databases without a patient identification number J Clin Epidemiol 2007 60 9 883 891 17689804 10.1016/j.jclinepi.2006.11.021

[B32] Tromp M Reitsma JB Ravelli AC Meray N Bonsel GJ Record linkage: making the most out of errors in linking variables AMIA Annu Symp Proc 2006 2006 779 783 17238447 PMC1839331

[B33] The International Statistical Classification of Diseases and Related Health Problems, Australian Modification – Tabular List of Diseases and Alphabetic Index of Diseases http://nccc.uow.edu.au/icd10am/icd10am/index.html

[B34] Australian Bureau of Statistics. Socio-economic Indexes for Areas (SEIFA), Data only 2006 Catalogue 2033.0.55.001 http://www.abs.gov.au/ausstats/abs@.nsf/mf/2033.0.55.001/

[B35] Taylor LK Travis S Pym M Olive E Henderson-Smart DJ How useful are hospital morbidity data for monitoring conditions occurring in the perinatal period? Aust N Z J Obstet Gynaecol 2005 45 1 36 41 10.1111/j.1479-828X.2005.00339.x 15730363

[B36] Validation Study NSW Midwives Data Collection 1998. NSW Mothers and Babies 1998 State Publication No (EPI) 000029 2000 9 S-2 97 99 Sydney: NSW Public Health Bulletin. NSW Department of Health

[B37] Pym M Taylor L Validation study of the NSW Midwives Data Collection 1990. *State Publication No (EHSEB) 93-167 Sydney* NSW Public Health Bulletin 1993 4 S-8 1 6

[B38] SAS Institute Inc SAS 9.2 [computer program] 2008 Cary: SAS Institute Inc

[B39] Adams MM Kirby RS Measuring the accuracy and completeness of linking certificates for deliveries to the same woman Paediatr Perinat Epidemiol 2007 21 Suppl 1 58 62 17593198 10.1111/j.1365-3016.2007.00838.x

[B40] Adams MM Berg CJ McDermott JM Gaudino JA Casto DL Wilson HG McCarthy BJ Evaluation of reproductive histories constructed by linking vital records Paediatr Perinat Epidemiol 1997 11 1 78 92 10.1111/j.1365-3016.1997.tb00799.x 9018730

[B41] Gyllstrom ME Jensen JL Vaughan JN Castellano SE Oswald JW Linking birth certificates with Medicaid data to enhance population health assessment: methodological issues addressed J Public Health Manag Pract 2002 8 4 38 44 15156637 10.1097/00124784-200207000-00008

[B42] Ananth CV Oyelese Y Yeo L Pradhan A Vintzileos AM Placental abruption in the United States, 1979 through 2001: temporal trends and potential determinants Am J Obstet Gynecol 2005 192 1 191 198 10.1016/j.ajog.2004.05.087 15672024

[B43] Danel I Berg C Johnson CH Atrash H Magnitude of maternal morbidity during labor and delivery: United States, 1993-1997 Am J Public Health 2003 93 4 631 634 10.2105/AJPH.93.4.631 12660209 PMC1447802

[B44] Kuklina EV Whiteman MK Hillis SD Jamieson DJ Meikle SF Posner SF Marchbanks PA An enhanced method for identifying obstetric deliveries: implications for estimating maternal morbidity Matern Child Health J 2008 12 4 469 477 10.1007/s10995-007-0256-6 17690963

